# Pregnancy-Related Spinal Biomechanics: A Review of Low Back Pain and Degenerative Spine Disease

**DOI:** 10.3390/bioengineering12080858

**Published:** 2025-08-10

**Authors:** Ezra T. Yoseph, Rukayat Taiwo, Ali Kiapour, Gavin Touponse, Elie Massaad, Marinos Theologitis, Janet Y. Wu, Theresa Williamson, Corinna C. Zygourakis

**Affiliations:** 1 Department of Neurosurgery, Stanford University Medical Center, Stanford, CA 94305, USA; 2 Department of Neurosurgery, Massachusetts General Hospital, Harvard Medical School, Boston, MA 02115, USA; 3 Department of Neurosurgery, Perelman School of Medicine, University of Pennsylvania, Philadelphia, PA 19104, USA

**Keywords:** pregnancy biomechanics, low back pain, LBP, degenerative spondylolisthesis, pelvic tilt, lumbar lordosis, spinopelvic parameters

## Abstract

Pregnancy induces substantial anatomical, hormonal, and biomechanical changes in the spine and pelvis to accommodate fetal growth and maintain postural adaptation. This narrative review synthesizes peer-reviewed evidence regarding pregnancy-related spinal biomechanics, with a particular focus on low back pain, spinopelvic alignment, sacroiliac joint dysfunction, and potential contributions to degenerative spinal conditions. A systematic search of PubMed, Embase, and Google Scholar was conducted using Boolean operators and relevant terms, yielding 1050 unique records, with 53 peer-reviewed articles ultimately cited. The review reveals that increased lumbar lordosis, ligamentous laxity, altered gait mechanics, and muscular deconditioning elevate mechanical load on the lumbar spine, predisposing up to 56% of pregnant individuals to low back pain. These changes are often associated with sacroiliac joint laxity, anterior pelvic tilt, and multiparity. Long-term risks may include degenerative disc disease and spondylolisthesis. Conservative interventions such as pelvic floor muscle training, prenatal exercise, and surface topography monitoring offer symptom relief and support early rehabilitation, although standardized protocols and longitudinal outcome data remain limited. Pregnancy-related spinal changes are multifactorial and clinically relevant; an interdisciplinary approach involving spinal biomechanics, physical therapy, and obstetric care is critical for optimizing maternal musculoskeletal health.

## 1. Introduction

Women undergo significant anatomical and physiological changes during pregnancy to meet the extensive demands required for fetal development and childbirth. These changes include musculoskeletal adaptations, such as accommodating increased body mass index (BMI) and pelvic adjustments during delivery. Past studies have examined the association between gravidity or parity and biomechanical alterations to the spine during pregnancy, hypothesizing that these changes may be associated with future back pain or degenerative spine disorders. Understanding these biomechanical spinal changes is crucial, as up to 56% of women experience low back pain during pregnancy, significantly impacting quality of life [1,2].

While the biomechanical changes in pregnancy have been individually described, their integration into a broader understanding of long-term spinal degeneration, including conditions like spondylolisthesis and disc degeneration, remains poorly defined. Few studies synthesize how hormonal, postural, and neuromuscular factors intersect to influence the onset or exacerbation of spinal pathology in postpartum populations. Interest in pregnancy-related spinal biomechanics has evolved considerably since the 1980s. Earlier investigations largely focused on pain prevalence and obstetric ergonomics, while more recent research has incorporated gait analysis, spinal alignment modeling, and hormonal profiling. This temporal shift reflects advances in biomechanics methodology and growing awareness of pregnancy as a complex state of mechanical adaptation. By recognizing this progression, our review contextualizes the current literature within the broader scientific evolution of the field.

This narrative review consolidates the current literature on pregnancy-induced spinal adaptations and their potential contribution to spinal degeneration. We examine alterations in spinopelvic parameters, discuss mechanisms underlying pregnancy-related LBP, and evaluate conservative interventions aimed at prevention and symptom mitigation. By bridging biomechanics with clinical implications, our aim is to support a more comprehensive understanding of pregnancy’s impact on spinal health and inform multidisciplinary approaches to prenatal musculoskeletal care.

## 2. Methods

### 2.1. Study Design

This review employed a narrative review methodology to synthesize the literature on spinal biomechanics and low back pain related to pregnancy. A narrative review was chosen for its suitability in integrating diverse types of clinical, biomechanical, and anatomical evidence, particularly when heterogeneous methodologies or outcome measures across studies preclude formal meta-analysis. This approach enabled a comprehensive understanding of anatomical adaptations, spinopelvic parameters, sacroiliac joint pain, and related conservative interventions. Literature searches were conducted across three databases: PubMed, Embase, and Google Scholar, without date restrictions, with the last search conducted in March 2025. Boolean operators (“AND”, “OR”) were used to combine search terms including the following: “pregnancy biomechanics,” “spinopelvic parameters,” “sacroiliac joint pain,” “lumbar lordosis,” “low back pain,” and “degenerative spondylolisthesis.” No restrictions were placed on publication year to incorporate foundational studies in spine biomechanics and pregnancy-related musculoskeletal research. As such, landmark studies from the 1980s–1990s were included due to their enduring relevance to modern spine biomechanical understanding. This narrative review was conducted in accordance with the JBI Critical Appraisal Checklist for Narrative, Expert Opinion and Text. The checklist was used to guide assessment of source credibility, analytical rigor, and peer-supported interpretation in line with best practices for narrative evidence synthesis.

### 2.2. Search Strategy

**Figure 1:**
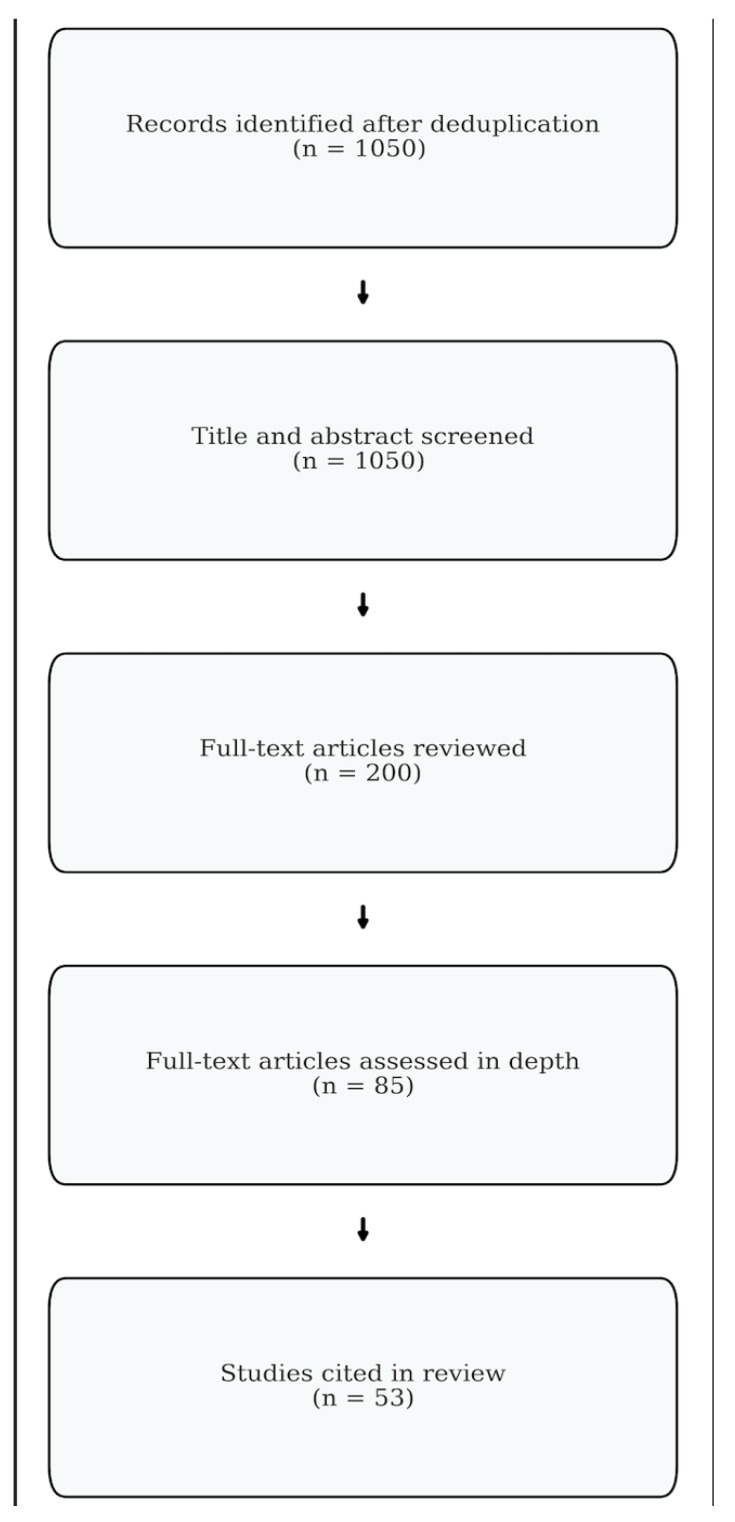
Methods flowchart. Diagram illustrating the identification, screening, and inclusion process of articles reviewed in this narrative synthesis.

A total of 1050 records were identified after duplicate removal across databases. Initial screening by title and abstract yielded 200 articles for full-text assessment (Figure 1). Studies were included if they were peer-reviewed human studies published in English; focused on spinal, spinopelvic, or sacroiliac adaptations during or after pregnancy; evaluated musculoskeletal, biomechanical, diagnostic, or non-surgical therapeutic approaches; and included clinical relevance or implications for spinal care. Exclusion criteria included animal studies, surgical-only outcomes, reviews lacking original data, and non-English or unpublished sources such as books, theses, and conference abstracts. Narrative synthesis was used to organize findings into key domains: anatomical and physiological adaptations, biomechanical compensation, low back and sacroiliac joint pain etiologies, degenerative spine risk, and conservative management strategies. Given the descriptive nature of this review, no formal risk-of-bias or quality assessment was performed. However, only peer-reviewed, full-length articles were included to enhance consistency and reliability. Reference management and duplicate removal were conducted using Zotero (Version 6).

### 2.3. Study Selection

The final manuscript includes 53 cited studies. These citations were selected from a larger pool of approximately 85 full-text articles that were reviewed for relevance and thematic contribution. Not all full-text studies were directly cited in the manuscript, as only those providing central or representative data were retained.

## 3. Results

### 3.1. Anatomic and Physiologic Changes During Pregnancy

The long-lasting impact of pregnancy on the spine and pelvis can vary widely among individuals, with several factors contributing to this heterogeneity, including a woman’s lifestyle before and during pregnancy, the size and position of the fetus, or even pre-existing spinal and musculoskeletal conditions. Research on the long-lasting effects of pregnancy on the spine has investigated how vaginal delivery and parity can impact the spinopelvic parameters. One study of 320 women who underwent whole-spine radiographs showed that pelvic incidence and sacral slope were significantly greater in women who delivered vaginally compared to those who delivered by C-section, while both of them significantly increased with the number of vaginal deliveries [3]. However, vertebral body dimensions remain unaffected, as evidenced by a population-based middle-aged birth cohort study by Oura et al., revealing no association between gravidity or parity and vertebral cross-sectional area and height ratio [4].

Pregnancy induces a range of physiological and anatomical adaptations designed to accommodate fetal growth while maintaining maternal function. Among the earliest and most pronounced changes is an increase in joint laxity, mediated largely by hormonal influences such as relaxin and estrogen. Calguneri et al. (1982) demonstrated measurable increases in ligamentous flexibility in pregnant women, particularly during the second and third trimesters, highlighting the body’s preparation for parturition through altered connective tissue integrity [5]. This generalized increase in joint laxity, while adaptive, may compromise the mechanical stability of load-bearing joints, including the spine and pelvis.

These hormonal changes intersect with biomechanical demands as maternal weight and abdominal girth increase. Hartmann and Bung (1999) emphasize the importance of addressing these changes through physical conditioning, noting that insufficient neuromuscular control and core strength may exacerbate postural imbalances and spinal strain [6]. Similarly, Artal and O’Toole (2003) underscore the need for structured guidelines surrounding peripartum exercise to prevent injury and maintain spinal health [7]. Their recommendations suggest that proper musculoskeletal conditioning may mitigate the risk of pain and dysfunction in late pregnancy and the postpartum period.

The hormonal driver relaxin plays a critical role in modifying ligamentous properties. Dehghan et al. (2014) explored its systemic effects, reporting that increased relaxin levels can reduce the tensile strength of ligaments, further increasing the susceptibility to pelvic instability and sacroiliac dysfunction [8]. Taken together, these studies indicate that pregnancy-related changes in laxity, muscle function, and posture must be understood not as isolated phenomena, but as an integrated response with direct biomechanical implications for spinal load distribution and compensatory mechanisms.

As pregnancy progresses, there is also a notable increase in body mass, along with a significant expansion of the abdominal girth, with weight gain ranging between 5 and 18 kg, depending on the woman’s BMI before pregnancy [9,10]. Several studies have examined how increases in body mass impact spinopelvic parameters, with Pauk and Swinarska reporting that maternal BMI was significantly associated with increased thoracic kyphosis in late pregnancy (R = 0.50, *p* < 0.05) [11]. Contrastingly, a different cross-sectional study demonstrated that there was a poor correlation between different BMI categories and spinopelvic values or lumbar lordosis types. When they categorized non-pregnant patients according to the presence and absence of obesity, the spinopelvic parameters increased in the obese group but did not reach statistical significance. Similarly, in order to investigate how fat distribution influences spinal anatomy, the researchers made a dichotomous comparison between patients with normal and elevated abdominal circumference, but they did not find a statistically significant correlation between abdominal obesity and frequency of lumbar lordosis [12]. This disparity ultimately suggests that pregnancy involves additional hormonal and musculoskeletal adaptations, which further influence spinal alignment and spinopelvic balance. Ultimately, these findings underscore the need to view pregnancy-related biomechanical changes not as isolated structural phenomena, but as part of a hormonally mediated continuum that informs both spinal vulnerability and the rationale for targeted clinical interventions.

### 3.2. Biomechanical Changes During Pregnancy

**Figure 2:**
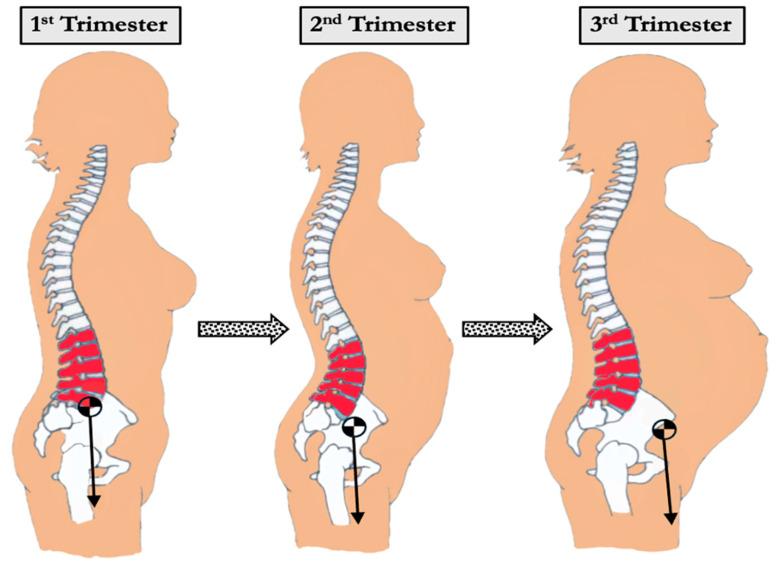
Changes in center of mass shift during pregnancy progression. An increase in abdominal mass during pregnancy shifts the center of mass forward, leading to mechanical strain.

Pregnancy induces dynamic and multifaceted biomechanical changes in the spine and pelvis to accommodate fetal growth, hormonal fluctuations, and shifting weight distribution. These adaptations are essential for maintaining postural stability and upright gait but can increase mechanical stress on the spine and contribute to musculoskeletal discomfort (Table 1). Developing a comprehensive understanding of the biomechanical changes in the spine during pregnancy may allow for understanding pathologies, including chronic low back pain, which pose a significant burden to patients in their daily lives. In describing the evolution of the spine, Whitcome et al. highlight several adaptations of the Hominin axial skeletons that support bipedalism, including elongated lumbar regions in both the number and length of the vertebrae as well as the marked posterior concavity of wedged lumbar vertebrae called lordosis [13]. This lordotic curvature functions to stabilize the torso over the pelvis and counteracts anterior displacement of the center of mass (Figure 2). During pregnancy, this forward shift in mass requires compensatory spinal realignment to prevent postural decompensation and mechanical overload.

**Figure 3:**
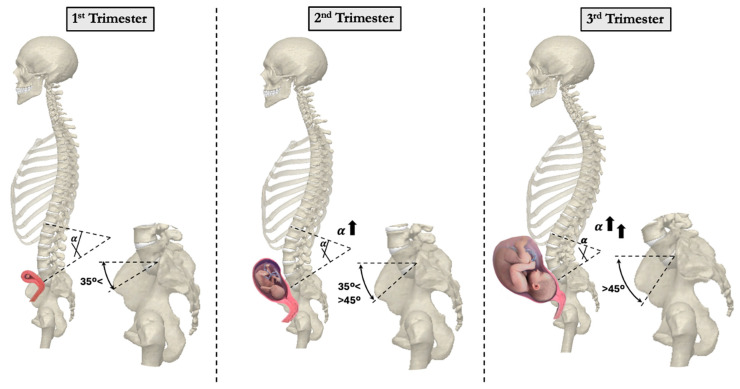
Increased lumbar lordosis and sacral slope during pregnancy progression. Changes in spinopelvic parameters, such as lumbar lordosis and sacral slope, occur throughout pregnancy. These adaptations maintain balance and mobility but may also contribute to musculoskeletal discomfort and long-term spinal degeneration.

**Figure 4:**
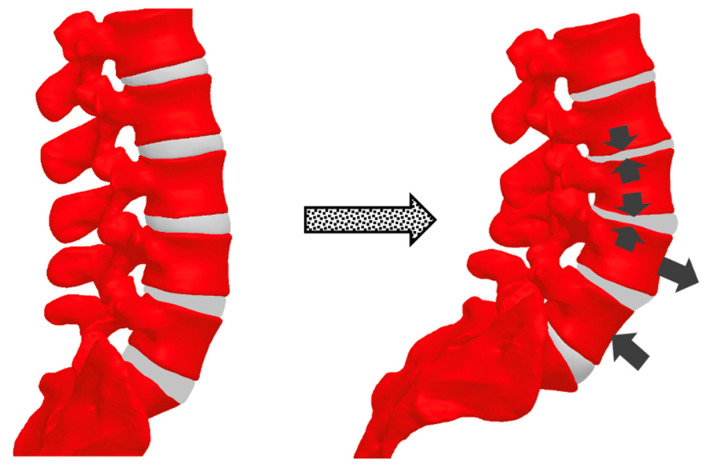
Bulging disc secondary to increased load and lumbar lordosis during pregnancy. Compensatory changes in spinopelvic parameters may predispose individuals to degenerative conditions such as intervertebral disc degeneration, leading to chronic back pain.

As pregnancy progresses, increasing body mass and anterior abdominal expansion further accentuate spinal curvature. One adaptive mechanism is the exaggeration of lumbar lordosis to maintain spinal alignment above the pelvis and preserve upright posture (Figure 3). Studies comparing pregnant women during the 2nd and 3rd trimesters with non-pregnant women found increased curvature of both the thoracic and lumbar spine [14]. Thoracic kyphosis increased from 10.7 degrees in the 2nd trimester to 11.5 degrees in the 3rd trimester. Similarly, lumbar lordosis increased from 9.0 degrees in the 2nd trimester to 10.0 degrees in the 3rd trimester, while non-pregnant women had an average lumbar lordosis of 7.3 degrees. Another study using 3D measurements with surface topography found a 7.4-degree change in thoracic kyphosis angle and an 8.4-degree change in lumbar lordosis angle in a uniparous pregnant woman from the 17th to the 37th week of pregnancy [22,23]. Whitcome et al. revealed that women at full term extend their lower back by nearly 60%, from a mean angle of 32 ± 12° in early pregnancy to 50 ± 12° at term [13]. These spinal adjustments, while protective, may increase load on the facet joints and intervertebral discs, potentially contributing to the development of degenerative pathology (Figure 2 and Figure 4).

In addition to spinal curvature changes, pregnancy introduces biomechanical challenges to postural stability. As gestation progresses, the anterior shift in the center of mass requires neuromuscular compensation to maintain balance. This adaptation is reflected in center of pressure (CoP) displacement during static stance, with studies showing a consistent anterior shift in CoP and increased sway area in pregnant individuals compared to non-pregnant controls, changes indicative of reduced postural control [9]. These balance alterations may be associated with the development or exacerbation of low back pain, though individual variability in compensatory strategies appears substantial. Dynamic assessments show that postural stability declines further in late pregnancy, particularly during gait initiation and transitions. In a motion analysis study, McCrory et al. demonstrated that women in their third trimester exhibited poorer dynamic balance scores and delayed neuromuscular responses during walking compared to non-pregnant controls, suggesting increased instability during daily activities [24]. Gait analysis studies have produced mixed findings; while some report no significant changes in walking velocity or stride length, others have observed reduced velocity, wider stride, and altered joint kinematics, including increased hip flexion and reduced ankle plantarflexion [16,17,25]. These discrepancies are likely due to individual differences in trunk control and neuromuscular adaptation, as supported by Krkeljas, who found that reduced trunk stiffness and compensatory gait changes were common responses to gestational biomechanical demands [26]. These findings illustrate that pregnancy significantly affects both static and dynamic balance mechanisms, contributing to the multifactorial etiology of low back pain and underscoring the need for individualized assessment and preventive intervention. In summary, these postural and neuromuscular adjustments though protective in nature appear to impose a cumulative mechanical load that may underlie the increased prevalence of musculoskeletal discomfort and spinal pathology in later pregnancy stages or postpartum.

### 3.3. Etiology of Pregnancy-Related Back Pain

Lower back pain typically begins in the second trimester and is the most common musculoskeletal complaint in pregnancy, affecting up to 56% of pregnant individuals [9]. Low back pain during pregnancy can be up to four times worse than in non-pregnant women, significantly impacting day-to-day functioning and quality of life [22,23]. However, these symptoms are not isolated to the pregnancy period and can translate to chronic postpartum disability. The prevalence of postpartum low back pain is reported to be as high as 5–43% at six months postpartum and 20% at three years after delivery [14,27]. Furthermore, 15% of women with chronic low back pain report initial onset during pregnancy. Pregnancy-related lower back pain is defined by pain in the lumbar region, is dull in character, and is often elicited by forward flexion. It is also characterized by the restriction of spine movement in the lumbar region with worsening pain on palpation of erector spinae muscles [28].As expected, lower back pain in pregnancy can significantly impact quality of life and may have socioeconomic consequences due to work absenteeism [29]. It is important to distinguish between lumbar-origin and sacroiliac joint-related pain, the latter of which is also highly prevalent during pregnancy and may present similarly but require distinct management strategies [30].

The pathophysiological causes of lower back pain in pregnancy are multifactorial and include mechanical strain, pelvic ligamentous laxity, and vascular compression, which will each be discussed in further detail. Risk factors for lower back pain include pregestational history of back pain, back pain during prior pregnancies, and advanced maternal age. The enlarging uterus during pregnancy also leads to weakening of abdominal muscles and reduced muscle tone, which places additional strain on compensatory lumbar musculature [21]. This weakening may be due to mechanical stretch, neuromuscular inhibition, and impaired recruitment of core stabilizers like the transversus abdominis and multifidus [31]. It is estimated that about half of weight gain during pregnancy is gained in the abdomen [29]. The enlarging abdomen necessitates postural compensations such as increased lumbar lordosis and forward pelvic tilt. In addition to the weakening of lumbopelvic stabilizing musculature, these biomechanical alterations lead to posterior chain loading on the lower back [18].

**Figure 5:**
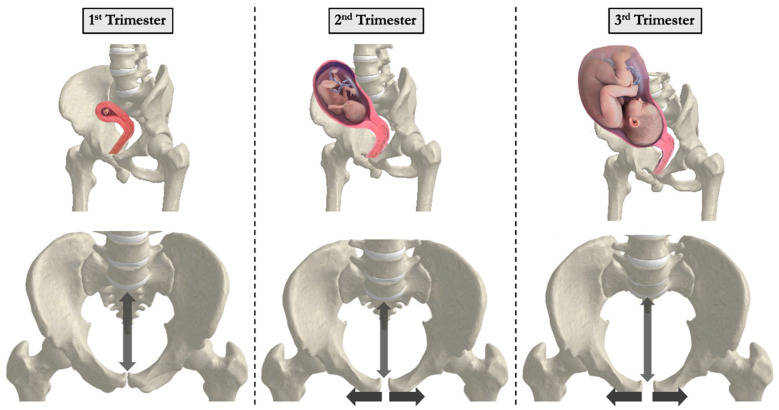
Widening of the pelvis and pubic symphysis during pregnancy. An increase in hormone-mediated joint laxity during pregnancy allows for the widening of the pubic symphysis to accommodate for childbirth. These changes have been implicated in lower back pain.

Sacroiliac (SI) joint pain is a distinct and highly prevalent contributor to pregnancy-related low back pain, often misattributed to lumbar spine pathology. It is typically localized just below the posterior superior iliac spine and may radiate into the buttocks or thighs, differing from the more central presentation of lumbar-origin pain. SI joint dysfunction is driven largely by hormonal ligamentous laxity and pelvic asymmetry, particularly during the later trimesters. Accurate clinical differentiation is important, as the underlying mechanisms and optimal treatment strategies differ from those used for lumbar spine conditions. Joint laxity increases during pregnancy to allow for the passage of the fetus. This has been attributed to the pregnancy hormone relaxin, with estrogen and progesterone as contributing factors [21,32]. The increase in joint laxity of the pubis symphysis has been implicated in lower back pain (Figure 5) [20].

Despite the well-documented biomechanical changes caused by the gravid uterus, the relationship between pregnancy-related weight gain and low back pain remains unclear in the literature. A retrospective study by Matsuda et al. examining the relationship between pregnancy-related weight gain and low back pain found that women reporting moderate-to-severe back pain at four months after delivery had 10.4 kg gestational weight gain compared to 9.0 kg in women without back pain [27]. Contrastingly, Boarg-stein et al. found no relationship between pregnancy-related low back pain and weight of the mother, weight gain during pregnancy, or weight of the baby [18].

The emerging literature suggests that vascular factors may also contribute to the pathogenesis of pregnancy-related low back pain. Fast et al. reported that nocturnal back pain was higher in pregnant women who exhibited decreased basal oxygen saturation and spent longer in the supine position. This finding supports the theory that compression of the inferior vena cava by the gravid uterus leads to venous stasis, reduced cardiac output, and congestion in the lumbar venous plexus. Compromised metabolic supply and edema in the lumbosacral neural microcirculation may result in pain, which in some patients is mitigated by adequate collateral circulation [19]. These findings underscore the importance of interventions such as compression stockings and regular ambulation to prevent pregnancy-related back pain (Table 2). Compression stockings enhance blood circulation, reducing venous stasis and associated discomfort, while regular movement prevents prolonged pressure on the inferior vena cava, which mitigates venous congestion [33]. These multifactorial contributors, ranging from anatomical and vascular to hormonal, likely interact in complex ways rather than act in isolation. For instance, hormonal laxity may potentiate musculoskeletal strain from rapid abdominal growth, while vascular compression could amplify nociceptive signaling, together exacerbating the subjective experience of pain. Building upon these findings, the following Section 3.4 and Section 3.5 explore a range of intervention strategies, including neuromuscular training, postural stabilization, and pain management, that address the biomechanical disruptions identified above.

### 3.4. Degenerative Spine Disease

The relationship between parity, gravidity, and spinal degeneration remains debated, with studies reporting both protective and deleterious associations depending on age, number of pregnancies, and anatomical focus. A 1996 study by Sanderson and Fraser found that the incidence of degenerative spondylolisthesis was significantly higher in multiparous women compared to nulliparous women and men, suggesting that repeated pregnancies may contribute to spinal instability through mechanical or hormonal mechanisms [42]. More recently, Cholewicki et al. identified parity and hysterectomy as independent predictors of degenerative spondylolisthesis in older women, even after adjusting for age and BMI [43]. Proposed contributing factors include increased joint laxity from hormonal shifts, cumulative axial loading of the spine during pregnancy, and weakening of abdominal musculature due to mechanical stretch and neuromuscular inhibition. These findings suggest that repeated pregnancies may disrupt spinal stability not only via direct mechanical strain but also through cumulative hormonal exposures that induce ligamentous laxity. Over time, this laxity, which is compounded by core muscle inhibition, may increase shear forces across lumbosacral joints, potentiating degenerative changes, particularly in predisposed individuals.

Conversely, a study by Çevik et al. reported that in women aged 20–40, increasing parity was associated with a lower Pfirrmann grade of disc degeneration, postulating that elevated estrogen levels during pregnancy may exert protective effects on intervertebral discs through receptor-mediated mechanisms [44]. This supports a broader view that hormonal shifts during pregnancy can play dual roles: protective in some anatomical contexts (e.g., intervertebral discs via estrogen receptors) yet deleterious in others (e.g., facet joint strain due to ligamentous laxity). Such differential effects may help explain contradictory findings in parity-degeneration associations across studies. A 2024 investigation by Güngör et al. found that higher parity (particularly five or more pregnancies) was associated with accelerated spinal degeneration, likely due to cumulative mechanical strain and altered hormonal regulation [45]. These mechanical and hormonal influences, especially increased lumbar lordosis, anterior mass loading, and ligamentous laxity, may contribute to intervertebral disc bulging or degeneration, particularly in the context of altered spinopelvic alignment (Figure 4). In this biomechanical context, the relative magnitude and persistence of lumbar lordosis may be key. While lordosis increases are often adaptive during pregnancy, exaggerated or poorly compensated curvature may elevate posterior joint compression and alter facet loading, promoting localized degenerative changes.

Changes in spinopelvic parameters are observed in patients with degenerative spondylolisthesis (DS). In a study of patients with lumbar spinal canal stenosis, both male and female patients who additionally had degenerative spondylolisthesis had significantly increased pelvic incidence (PI), sacral slope (SS), L4 slope, L5 slope, thoracic kyphosis (TK), and lumbar lordosis (LL). These parameters were also higher in two-level versus single-level DS patients, though comparison to non-DS controls was not made directly. Differentiating between single-level and two-level lumbar degenerative spondylolisthesis, these spinopelvic parameters were significantly larger in patients with two-level disease [15]. In another study on the pathogenesis of lumbar degenerative spondylolisthesis, weight was found to be a highly correlated factor, as the average body mass index (BMI) and percent of patients with BMI > 25 were significantly increased in the degenerative spondylolisthesis cohort [43,46]. Taken together with compensatory biomechanical changes during pregnancy, a theory for the pathogenesis of degenerative lumbar spondylolisthesis is that increased PI and LL are predisposing factors for the disease, and adding an overweight body constitution forces the body to compensate by decreasing PT (entering a “compensated balance” state), which then facilitates the development of degenerative disease (an “unbalanced” state) [46,47]. Parity has also been shown to be associated with significantly greater PI-LL and thoracic kyphosis, the first of which has been shown in studies detailed above to be correlated with lumbar degenerative spondylolisthesis. However, in this study, parity itself was not found to be directly correlated with lumbar disc/spine degeneration [48]. These theories suggest that the compensatory changes in spinopelvic parameters during pregnancy may be a predisposing factor to spine degeneration. Overall, the collective evidence indicates that spine degeneration in pregnancy is not solely dependent on parity or BMI, but rather emerges from a confluence of anatomical, hormonal, and postural changes. These interact dynamically, and their cumulative effect may render the spine increasingly susceptible to degenerative spondylolisthesis in a subset of individuals.

### 3.5. Treatment of Pregnancy-Related Lower Back Pain

Several studies have been conducted to understand how the risk of back pain associated with pregnancy can be minimized. For conservative management of pain, there is evidence suggesting maternity support belts and spinal manipulation are helpful with pain relief. Although chiropractic manipulative therapy (CMT) is gaining popularity for pregnancy-related back pain, evidence remains limited, and current guidelines recommend it as an adjunct rather than a primary treatment [49]. It is important to distinguish between interventions aimed at preventing pregnancy-related back pain and those intended to treat it once symptoms are present. Preventive strategies tend to emphasize biomechanical conditioning and postural stabilization, while treatment strategies often target symptom management and functional mobility. The strongest evidence suggests prenatal exercise and acupuncture are best for preventing pregnancy-related back pain. Prenatal exercise interventions, including stability ball training and aquatic-based exercise programs, activate the transverse abdominis and multifidus to enhance lumbopelvic stability and reduce pregnancy-related low back pain [29,48]. Beyond muscular strengthening, prenatal exercise likely confers benefits through neuromuscular re-education, fostering more efficient motor patterns during pregnancy-related biomechanical shifts. In addition, prenatal exercise is thought to prevent pregnancy-related weight gain within the recommended range, which may help mitigate lower back pain. Acupuncture has been found to be particularly effective at treating pelvic girdle pain, explaining its efficacy in back pain prevention.

Beyond standard prenatal fitness guidelines discussed above, targeted exercise interventions can also help bolster lumbopelvic stability and help mitigate pregnancy-related low back pain [34,38]. Pelvic floor muscle training (PFMT) including “Kegels” has been shown to have measurable benefits for pelvic stability along with reducing urinary incontinence and pelvic discomfort [35,36]. A meta-analysis of 11 randomized controlled trials involving 2347 pregnant women found that exercises, including pelvic floor strengthening and aerobics, reduced the risk of pregnancy-related LBP by 9% [50]. Deep core activation exercises act on the transverse abdominis to counteract trunk instability and have been shown to lower the risk of persistent low back symptoms [37,51]. For patients with sacroiliac joint dysfunction, pelvic stabilization exercises, targeted physical therapy, and sacroiliac belts have demonstrated effectiveness in reducing SI-related pain and improving functional mobility during pregnancy [52]. Prenatal yoga offers low-impact flexibility, balance, and postural exercises, with systematic reviews pointing to enhanced overall musculoskeletal function and comfort in pregnant populations. Yoga is thought to reduce pain during pregnancy by stimulating pressure receptors, which enhance vagal activity, lower cortisol and substance P, increase serotonin, and ultimately decrease stress, blood pressure, and the risk of pregnancy complications [53]. Yoga’s effects, including hormonal, postural, and autonomic regulation, position it as a uniquely integrative modality, aligning physical and psychological benefits during pregnancy. Altogether, these targeted programs provide a comprehensive approach for reinforcing core and pelvic floor musculature, potentially diminishing biomechanical stresses that accompany pregnancy.

Pharmacological management of lower back pain during pregnancy is challenging due to health considerations of the developing fetus. Pain-relieving medications such as non-steroidal anti-inflammatory drugs (NSAIDs) are contraindicated late in pregnancy due to their teratogenic effects, while acetaminophen and the muscle relaxant cyclobenzaprine are usually considered safe to use throughout pregnancy [39,40]. Epidural steroid injection is typically only indicated when there is evidence of lumbar nerve compression and spine surgery is not typically indicated for pain during pregnancy unless the pain renders the patient incapacitated or with a neurologic deficit [28]. Surface topography studies, which are non-invasive imaging techniques using optical scanning systems to assess spinal curvature, represent a viable alternative to radiation-based imaging and can help monitor spine curvature and postural changes throughout pregnancy [23,41]. These techniques may be especially valuable in detecting early maladaptive compensations in spinal curvature or pelvic tilt that precede the onset of pain, thus enabling preemptive intervention. These minimally invasive methods could become routine in prenatal care, potentially preventing lower back pain and biomechanical changes that might lead to degenerative spine disease later in life.

## 4. Future Directions

Future studies examining back pain during pregnancy should focus on recruiting a more representative patient sampling, as many of the present studies lack heterogeneous patient populations. Understanding how the spine is affected during pregnancy for a more diverse patient population will make the results gleaned from these studies more generalizable. Additionally, there is a need for more longitudinal studies that focus on the long-term impact of pregnancy-related spinal changes. Most of the existing literature emphasizes acute pain and treatments during pregnancy, with fewer studies investigating chronic pain and its management in the postpartum period.

Exploring the use of non-invasive methods like surface topography to monitor spine curvature and postural changes throughout pregnancy could yield valuable insights. These technologies might offer a preventive strategy for both pregnancy-related back pain and degenerative spine disorders later in life. Another exciting avenue is the use of artificial intelligence (AI)-driven predictive models to create large datasets, including patient demographics, pre-pregnancy spinal imaging, gait analysis, and physiological changes, to forecast musculoskeletal complications such as low back pain and degenerative spinal disease. Using AI modeling can help stratify risk factors and personalize preventive strategies, such as targeted prenatal exercise programs or early postural interventions.

## 5. Limitations

Several studies investigating the spine during pregnancy are limited in generalizability due to the homogeneity of their sampled populations. Historical and ongoing medical mistrust may contribute to underrepresentation of marginalized groups in pregnancy-related research, limiting the applicability of findings to diverse patient cohorts. Additionally, many of the studies cited in this review are retrospective or observational in nature, inherently limiting the ability to draw causal inferences. Finally, biomechanical factors during pregnancy are difficult to isolate due to the influence of co-occurring variables such as hormonal fluctuations, psychosocial stressors, and varying levels of physical activity, all of which may contribute to pregnancy-related back pain.

## 6. Conclusions

Ethical considerations are particularly important in this field, as the safety of both mother and fetus must be prioritized. Imaging techniques such as CT and X-rays raise concerns about fetal harm from radiation exposure, making non-invasive alternatives preferable. Another important consideration is the informed consent process. Pregnant individuals may face additional stress or anxiety due to the nature of their condition, and it is essential that researchers ensure that participants fully understand the risks, benefits, and purposes of the studies in which they are participating. The autonomy of pregnant participants must be respected, particularly in vulnerable or high-risk pregnancies where added pressures may complicate decision-making.

Understanding the biomechanical changes that occur in the spine during pregnancy is crucial for improving maternal health and preventing long-term musculoskeletal issues for half of our world’s population. Pregnancy induces significant anatomical and physiological adaptations, particularly in the spine and pelvis, which can lead to discomfort and increase the risk of conditions such as low back pain and degenerative spine disorders. This review emphasizes the importance of addressing these changes through early, non-invasive monitoring (e.g., surface topography) and preventive strategies such as targeted prenatal exercise. However, the existing literature is limited by homogeneous sampling and a lack of long-term follow-up, highlighting the need for more inclusive and longitudinal research. Advancing this understanding will enable earlier, more personalized interventions to support spinal health throughout pregnancy and beyond.

## Figures and Tables

**Table 1:** Key biomechanical changes during pregnancy.

Biomechanical Change	Mechanism	Clinical Significance	References
Increased Lumbar Lordosis	Forward shift in the center of mass and compensatory hyperextension of the lower spine	May elevate facet joint loads; can predispose to low back pain and spondylolisthesis	Whitcome et al., 2007 [13]; Yoo et al., 2015 [14]
Altered Pelvic Parameters (↑ Sacral Slope, ↑ Pelvic Tilt)	Hormonal relaxation of ligaments and change in weight distribution lead to greater pelvic incidence and sacral slope	Excessive pelvic tilt alters lumbopelvic biomechanics; may contribute to postpartum spinal issues and degenerative changes over time	Yamada et al., 2021 [3]; Wang et al., 2016 [15]
Changes in Gait and Balance	Abdomen enlargement shifts center of gravity anteriorly, altering stride length, cadence, and stance width	Altered balance can increase mechanical load on lower spine and pelvis; may cause postural instability and higher fall risk	Foti et al., 2000 [16]; Wu et al., 2004 [17]
Reduced Lumbopelvic Stabilization	Abdominal expansion reduces stability of transverse abdominis, multifidus, and pelvic floor	Reduced spinal support increases stress on lumbar structures, potentially aggravating low back pain	Fast et al. 1987 [1]; Borg-Stein et al., 2005 [18]; Fast et al. 1992 [19]
Pelvic Girdle and SI Joint Laxity	Elevated relaxin and progesterone levels lead to ligamentous laxity in the pubic symphysis and SI joints	Instability in the pelvic ring can co-occur with low back pain and is unique to pregnancy (not observed with non-pregnant weight gain)	Wu et al., 2004 [17]; Mens et al., 2009 [20]
Anterior Loading from the Growing Uterus	Progressive uterine and fetal enlargement places an anterior pull on the lower spine	Increases shear forces across the lumbar region; can amplify lordotic posture and contribute to degenerative disc or facet changes	Ritchie JR et al., 2003 [21]

**Table 2:** Evidence-based interventions for pregnancy-related low back pain.

Intervention	Mechanism	Evidence	References
Prenatal Exercise (PFMT, aquatics, stability ball, etc.)	Strengthens the deep core (transverse abdominis, multifidus) and pelvic floor (levator ani complex) while improving posture and offsetting excessive lumbar lordosis. Specific routines like aquatics and PFMT (“Kegels”) focus on lumbopelvic stability	Numerous RCTs and reviews link pelvic floor and core strengthening to reduced low back and pelvic pain, improved function, and better postural stability. Prenatal yoga/Pilates programs (with appropriate modifications) may also alleviate discomfort and reduce stress	Borg-Stein et al., 2005 [18]; Nascimento et al., 2012 [34]; Mørkved & Bø, 2014 [35]; Woodley et al., 2020 [36]; Wang XQ et al., 2012 [37]
Maternity Support Belts and Sacroiliac (SI) belts	Provides external support to the lower abdomen and lumbopelvic region. SI belts, a subtype of maternity belts, are specifically designed to stabilize the sacroiliac joints and control pelvic ring laxity. These supports reduce spinal load and improve functional mobility in patients with SI joint pain	Some trials indicate short-term pain relief and reduced postpartum pelvic pain, especially in cases of sacroiliac joint dysfunction. Evidence is variable, but SI belts are often included in conservative management	Casagrande et al., 2015 [29]; Mens et al., 2009 [20]
Acupuncture and Related Modalities	May modulate pain pathways and reduce pelvic girdle instability; believed to stimulate endorphin release and increase local blood flow	Cochrane reviews suggest beneficial effects for pelvic girdle and low back pain in pregnancy	Pennick & Liddle, 2013 [38]
Physical Therapy and Spinal Manipulation	Manual therapy techniques can address restricted spinal segments and muscular imbalances; guided exercises improve muscle activation	Mild to moderate relief in some pregnant populations; safety often considered good if performed by experienced clinicians	Ritchie, 2003 [21]; Borg-Stein et al., 2005 [18], Mens et al., 2009 [20]
Postural Re-education and Ergonomics	Teaches pregnant individuals to distribute weight more evenly, maintain neutral spine alignment, and use proper lifting mechanics	Anecdotally effective and often recommended, though high- quality RCT data may be sparse; widely included in comprehensive prenatal programs	Conder et al., 2019 [9]; Branco et al., 2014 [10]
Compression Stockings and Regular Ambulation	Reduces venous stasis in the inferior vena cava region (especially in 3rd trimester with prolonged standing) and improves blood flow to pelvic/lumbar tissues	Shown to alleviate nocturnal back pain associated with decreased basal oxygen saturation and supine positioning; may help reduce edema-related pain	Fast et al., 1992 [19]; Szkwara et al., 2019 [33]
Pharmacological Management	Limited safe options in pregnancy (e.g., acetaminophen, possibly muscle relaxants like cyclobenzaprine); opioids and NSAIDs often restricted by trimester-specific risks	Generally recommended only if pain is debilitating and after nonpharmacologic strategies; usage minimized for fetal safety	Black & Hill, 2003 [39]; Rathmell et al., 1997 [40]
Surface Topography and AI-Based Monitoring	Non-invasive imaging and predictive algorithms to track spinal curvature changes, posture, and potential LBP onset	Emerging data support early intervention if major postural alterations are detected; may reduce postpartum persistence of pain by guiding exercise	Michoński et al., 2016 [23], Betsch et al., 2015 [41]
